# The London School of Tropical Medicine

**Published:** 1903-12-12

**Authors:** 


### The London School of Tropical Medicine.

On the 7th inst., Sir Patrick Manson gave an address at the London School of Tropical Medicine on what the school has done, is doing, and hopes to do. The occasion was the departure of Sir Francis Lovell, the dean, for the East, on behalf of the school.

Sir Patrick Manson, in briefly recounting the history of the school, said that he would take advantage of the opportunity to make public acknowledgement of two obligations that those who were interested in the study and practice of tropical medicine laboured under. The first of these was to Mr. Chamberlain, of whom it might be truly said that he had been the mainspring in the movement for education in tropical medicine. The other acknowledgement was to the Seamen's Hospital Society and their governing body. Without them, so far as London was concerned, tropical medicine would have had no suitable habitation. Their intelligent appreciation of their responsibilities as administrators of a great public trust, their catholic interpretation of their duties could not have been better evidenced than by the support they had given to Mr. Chamberlain in his desire to foster tropical medicine.

Sir Patrick Manson then passed on to the two main objects of the London School of Tropical Medicine, namely, education and research. With regard to the former he stated that since the school had been opened on October 3, 1899, no fewer than 354 students had passed through its portals. During the first session there had been 27 students; their first two years they had had an aggregate of 149 students; their last two years they had had 178 students. The figures showed a steady increase in the numbers attending the school, but this increase in number did not adequately represent the increasing popularity and usefulness of the institution, for whereas in the earlier days of the school's existence many of the students came for one or two months only, latterly a large proportion of the students remained during the full course of three months. He felt justified in saying that every student who had entered at the school had had an opportunity of acquainting himself with the more important facts of tropical pathology, with the practical methods of diagnosing tropical disease, and with the practical methods of treating tropical disease. They had been in actual touch with the raw material, so to speak, the germ causes of disease, and the pathological effects of the operation of these germs on the human body. They had seen, and most of them had applied for themselves, the most recent methods of diagnosis. They had seen in operation the methods of treatment of best repute. It would be impossible to gainsay the value of such training. But, over and above this, their students in most instances had been sent out with a knowledge of the theory of the subject, thoroughly abreast of the times, knowing what had already been done, and thoroughly apprehending what still required to be done; in other words, they had gone forth qualified not only to practise the art, but also to advance the science of tropical medicine. So it had come about that a whole army of eager investigators had arisen, mainly composed of men educated or inspired by the Liverpool and London schools.

This multiplication of investigators, and therefore of chances of discovery, had already borne remarkable fruit. Without alluding to what their friendly rivals in Liverpool had done, he could point to not a few notable triumphs by men from the London School, although the latter had only been in existence a few months over four years. (1) He referred first of all to an observation made in their laboratory by their present superintendent, Dr. Low. For the first time he had shown in what manner the blood-worm, responsible for an important group of tropical diseases, represented by the hideous and disabling disease known as elephantiasis, gained access to the human body. He had shown that this worm at an early stage of its existence, like the germ of malaria, was introduced into the human tissues by the proboscis of the mosquito. This important discovery had a very practical bearing, for it indicated with precision the way by which elephantiasis and its congeners might be avoided. (2) Drs. Sambon and Low, sent by the school with the aid of funds partly supplied by the Colonial Office, had clearly demonstrated by an experiment that man can live in the midst of malaria if he only employs simple and practical measures against mosquito bites. These gentlemen, Signor Terzi, and their servants, lived for three or four months during the most unhealthy season of the year, in the most unhealthy place in Europe, and, thanks to the measures mentioned, not with injury, but with positive benefit to their health. (3) The school, by importing infected mosquitoes from Italy and then setting them to bite healthy individuals in their laboratory, and thereby promptly conferring malarial disease to those so bitten, had given the final and most telling proof up to that time obtained that malaria was conveyed by the mosquito. (4) A pupil of the school, Dr. Forde, who had learned there the value of the microscope in the diagnosis of tropical disease, had discovered in the Gambia Colony the presence in the blood of a man of a new parasite, a parasite which was subsequently recognised by Dr. Dutton, of the Liverpool School of Tropical Medicine, as belonging to a group of germs responsible for many and grave diseases among the lower animals. This discovery was already bearing remarkable fruit. (5) Only last year Dr. Castellani, another pupil of the School, had demonstrated the association of this same parasite, called a trypanosoma, with one of the most terrible diseases to which tropical humanity is liable—viz., sleeping sickness. Subsequent investigation by Colonel Bruce and others had wholly endorsed the truth and importance of Dr. Castellani's discovery.

There were other discoveries which might be accredited to the London School of Tropical Medicine, but those mentioned sufficed to show that the existence of the school had not been unproductive in that line, nor altogether in vain. That the few thousand pounds the establishment had cost the country had been more than well invested, who could gainsay? Those permanent additions to human knowledge, which as the years rolled on were bound to accumulate around them additional knowledge, would yield a huge compound interest, and do more than justify the school's existence.

He had already acknowledged their indebtedness to Mr. Chamberlain and to the Seamen's Hospital Society, but he would like to mention another name, that of Sir John Craggs, who had generously provided a scholarship of £300 a year, tenable for three years, for purposes of research. Since then he had made the school the recipients of a very handsome annual donation, to be awarded to any actual or past student of the school who should make an important contribution to tropical medicine.

However, the school's ambition to gather fresh laurels in the field of investigation was cramped. They wished to furnish their museum and their library on a scale adequate to the needs of the school. These, and many other requirements, demanded money. In short, they wanted £100,000, or as much of that sum as they could get. They had given full value for that money. Apart from the education they had supplied to their 354 students, any one of the discoveries mentioned above was worth the sum mentioned ten times over. They had given the public a great deal more than they had received, or were ever likely to receive. He had, therefore, no hesitation in asking for something more on account.

Two years ago, when they were in straits for funds, Sir Francis Lovell, their dean, paid a visit to the East, and succeeded in collecting for them a very substantial and very welcome sum. He was again about to leave on the same errand, and they were met that day to wish him God-speed in his self-imposed, somewhat arduous, and not very pleasant task.

